# Alfred Nobel and His Prizes: From Dynamite to DNA

**DOI:** 10.5041/RMMJ.10311

**Published:** 2017-07-31

**Authors:** Marshall A. Lichtman

**Affiliations:** Department of Medicine and the James P. Wilmot Cancer Institute, University of Rochester Medical Center, Rochester, NY, USA

**Keywords:** Alfred Nobel, Nobel Foundation, Nobel Prizes, Nobel Prize in Physiology or Medicine

## Abstract

Alfred Nobel was one of the most successful chemists, inventors, entrepreneurs, and businessmen of the late nineteenth century. In a decision later in life, he rewrote his will to leave virtually all his fortune to establish prizes for persons of any nationality who made the most compelling achievement for the benefit of mankind in the fields of chemistry, physics, physiology or medicine, literature, and peace among nations. The prizes were first awarded in 1901, five years after his death. In considering his choice of prizes, it may be pertinent that he used the principles of chemistry and physics in his inventions and he had a lifelong devotion to science, he suffered and died from severe coronary and cerebral atherosclerosis, and he was a bibliophile, an author, and mingled with the literati of Paris. His interest in harmony among nations may have derived from the effects of the applications of his inventions in warfare (“merchant of death”) and his friendship with a leader in the movement to bring peace to nations of Europe. After some controversy, including Nobel’s citizenship, the mechanisms to choose the laureates and make four of the awards were developed by a foundation established in Stockholm; the choice of the laureate for promoting harmony among nations was assigned to the Norwegian Storting, another controversy. The Nobel Prizes after 115 years remain the most prestigious of awards. This review describes the man, his foundation, and the prizes with a special commentary on the Nobel Prize in Physiology or Medicine.

## INTRODUCTION

Since the first Nobel Prizes were awarded in 1901, the recipients have captured the interest of the world’s scientific, literary, and political communities. In December, the prize winners in the categories of chemistry, physics, physiology or medicine, and literature are honored at a ceremony in Stockholm where they receive their diploma and medal, and a document indicating the value of their share of that year’s monetary award and deliver a lecture describing the significance of the work leading to the prize. After the awards ceremony they participate in a lavish banquet hosted by the Swedish royal family. Simultaneously, the Peace Prize is awarded by the Royal Norwegian Academy in Oslo, according to the directives of Alfred Bernhard Nobel’s will.

No other prize for contributions to humankind holds the same prestige.^1^,^2^ The recipients will be hailed in their institutes, communities, and countries. The science laureates will attract the most promising students to their laboratories. The laureates will be honored by political leaders. Their signatures on petitions and newspaper advertisements supporting political and social policy positions will be given weighty consideration. The prestige of the prize results in the laureates being claimed as products of all past institutions with which they were affiliated, even if the association had little or nothing to do with the achievements that led to the prize.

The more prestigious the institution the more fastidious is their claim to a Nobel Laureate’s achievement. Harvard, for example, does not make a claim regarding its alumni who subsequently received a Nobel Prize, only those who did the work for which the prize was awarded at Harvard. In contrast, the University of Rochester named a large, newly built medical research building for Arthur Kornberg in 1999, who shared the Nobel Prize in Physiology or Medicine with Severo Ochoa in 1959 for their description of the biological synthesis of ribonucleic and deoxyribonucleic acid. Kornberg received his MD degree at the University of Rochester in 1941. Neither the ideas nor the work that led to the prize were even conceivable at that time, given the limited state of knowledge of deoxyribonucleic acid (DNA), ribonucleic acid (RNA), and nucleotide biosynthesis. He was a medical student. Moreover, Kornberg harbored longstanding ill-will about his failure to be selected by Dean George Hoyt Whipple for a year-out research fellowship between the second and third year of medical school, which Kornberg attributed to his being Jewish, as detailed in his autobiography, *For the love of enzymes: The odyssey of a biochemist*.^3^ In any case, Harvard can be aloof about laureates who merely passed through its ivied halls; other institutions’ claims are less fastidious.

Through 2016, 115 years since the initiation of the prizes, the Nobel Foundation’s list of laureate affiliations had only one of 911 laureates shown as affiliated with the University of Rochester as the research site of his or her work leading to the prize. That person was George Whipple who shared the Nobel Prize in Physiology or Medicine in 1934, a matter to be discussed later in this essay. Another laureate, Henrik Carl Peter Dam, was at the time of the receipt of the Nobel Prize in Physiology or Medicine a senior associate in biochemistry at the University of Rochester.^4^ He shared the prize in 1943 with the American biochemist Edward Adelbert Doisy for the discovery of vitamin K. This vitamin is required for the complete formation of several proteins that participate in normal blood coagulation. The work was done at the University of Copenhagen by Dam and his wife. He left Denmark on a lecture tour in 1940 to the United States and Canada, just after the Nazis occupied Denmark, and he gained sanctuary in Rochester at the invitation of Dean Whipple. The award was made in New York City on December 10, 1943 by the Swedish Minister, Wollmar F. Bostroem, with King Gustav V sending his congratulations; the wartime conditions prevented travel to Stockholm.

## THE PRIZES

The fund that supports the prizes was derived from virtually all of Alfred Nobel’s assets and the sale of all properties and business holdings following his death from a cerebral hemorrhage in San Remo, Italy on December 10, 1896 at the age of 63 years.^5^–^9^ He also had coronary artery disease and angina pectoris for which he was treated with nitroglycerin, the essential chemical in the explosive he developed. In a letter to a friend he commented on this unusual coincidence: “Isn’t it the irony of fate that I have been prescribed nitroglycerin, to be taken internally! They call it Trinitrin, so as not to scare the chemist and the public.”^10^

He was a bachelor. Thus, a small fraction of his estate’s financial assets was divided among nieces and nephews, friends, and servants. In a short statement at the end of his instructions pertaining to family and friends, he requested that,

… the remaining realizable estate shall be dealt with in the following way: the capital, invested in safe securities by my executors, shall constitute a fund, the interest of which shall be annually distributed in the form of prizes to those who, during the preceding year, shall have conferred the greatest benefit on mankind. The said interest shall be divided into five equal parts, which shall be apportioned as follows: one part to the person who shall have made the most important discovery or invention in the field of physics; one part to the person who shall have made the most important chemical discovery or improvement; one part to the person who shall have made the most important discovery within the domain of physiology or medicine; one part to the person who shall have produced in the field of literature the most outstanding work of an idealistic tendency; and one part to the person who shall have done the most or best work for fraternity between nations, for the abolition or reducing of standing armies and for the holding and promotion of peace congresses. [The last-mentioned became known as the Nobel Prize for Peace.] The prizes for physics and chemistry shall be awarded by the Swedish Academy of Sciences; that for physiological or medical work by the Caroline Institute in Stockholm; that for literature by the Royal Swedish Academy in Stockholm, and that for champions of peace by a committee of five persons to be elected by the Norwegian Storting. It is my express wish that in awarding the prizes no consideration whatsoever shall be given to the nationality of the candidates …^11^

In 1968, as an act commemorating the 300th anniversary of the founding of the Bank of Sweden, a sixth prize was established, designated officially as “The Bank of Sweden Prize in Economic Sciences in memory of Alfred Nobel.” Although there was consternation about this intrusion on Nobel’s intentions, as described in his will, the prize has come to be accepted functionally by Sweden and the world as a sixth Nobel Prize. It was first awarded in 1969, and, through 2016, 48 awards to 78 laureates have been made.

## THE MAN

Nobel’s philanthropic interests may have crystallized after reading his own obituary in a Paris newspaper. Several newspapers in Paris erroneously reported his death, when it was his brother, Ludvig, who had died in 1888 in Cannes. Alfred was living in Paris. The Parisian newspaper mistook Ludvig for Alfred and reported Alfred’s supposed death with the headline, “*Le marchand de la morte est mort*” (“The merchant of death is dead”). The obituary and the media coverage he received during his career highlighted the manufacturing of explosive chemicals and devices and the development of armaments. He was portrayed as an arms merchant. The journalist who mistakenly published his obituary stated that Nobel “… became rich by finding ways to kill more people faster than ever before.”^5^

**Figure 1:**
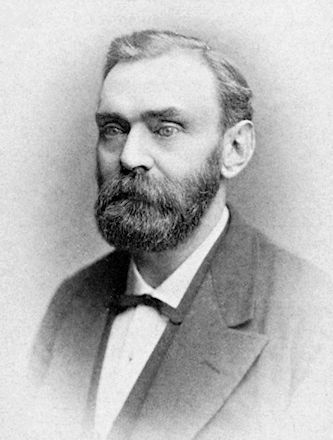
Portrait of Alfred Nobel.

Nobel was an accomplished chemist, inventor, entrepreneur, and industrialist. He became one of the most notable and wealthy men of the late nineteenth century (Figure 1). He was born in Stockholm in 1833. The Nobels were descended from a seventeenth-century Swedish physician, scientist, and scholar, Olof Rudbeck the Elder, who became the Rector of the University of Uppsala.^12^ Rudbeck’s daughter married Peter Olai Nobelius. It was from this marriage that the Nobel family descended. (Nobel is the shortened form of Nobelius, a Latinized habitational name from the village of Nöbbelöv.)

Immanuel Nobel, Alfred Nobel’s father, planned to build a canal at Suez, resulting in his interest in explosives. The methods for such enormous building projects were those used by the Romans. These projects would benefit from explosives that were capable of displacing large amounts of rock. Also, the Russian military was intrigued by his experiments with explosives and requested that he develop two systems that had military applications: land mines to defend army bases or towns, and sea mines to protect harbors and docked ships. The Russian army used his products, and he gained economic security and established his home in St Petersburg.

At age 8 years, Alfred Nobel moved to St Petersburg with his parents and brothers. He was tutored in the sciences and the humanities there, but never matriculated in a school or received a degree. Alfred’s diverse interests and ingenuity were so impressive that at age 17 years his father sent him to France, Germany, Italy, and the United States, studying chemistry and conferring with chemists and industrialists to gain technical information that would enhance their businesses.^5^–^7^ He had developed a fluency in French, German, Italian, English, Swedish, and Russian. Alfred returned to St Petersburg in 1852, at the age of 19. The Nobel enterprise grew because of the demand for munitions and armaments to support Russia’s participation in the Crimean War, from 1853 to 1856.

Thereafter, the Nobels, collaborating with the Rothschilds, used Russian oil to end Standard Oil’s and John D. Rockefeller’s monopoly on the world’s oil supply.^13^ Standard Oil had early success as a result of the conversion of crude oil to kerosene, the flammable that replaced whale oil for lamps, the principal form of lighting at the time. The Nobel and Rothschild families’ development of Russian oil intruded on Standard Oil’s control of world markets.

The territory around Baku near the Caucasus Mountains in what is now Azerbaidzhan had been known as the region of eternal fire. Gas escaping from superficial underground oil deposits ignited spontaneously, and this “eternal flame” was the basis for the Zoroaster religion. The industry developing in Russia near Baku used innovations in oil drilling techniques developed in the United States.

**Figure 2:**
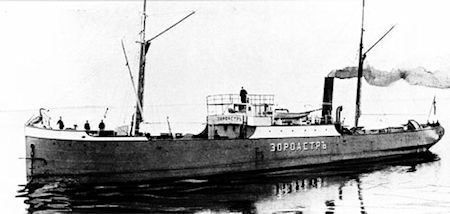
The First Oil Tanker, the Zoroaster, Built in 1878. The hull was built with steel, which became available in the last half of the nineteenth century as a result of Henry Bessemer’s development of a method to produce large quantities of steel from pig iron. The ship contained iron tanks to hold the oil. This innovation was the brainchild of Ludvig and Robert Nobel. They developed an oil pipeline to carry the oil from its source to the ship’s tanks. They developed a system of ballast to stabilize the ship. They subsequently built additional tankers to carry oil across the Caspian Sea and up the Volga and Don rivers. The ships, built in Sweden, could reach the Caspian Sea by sailing the Baltic Sea, the canals, and smaller rivers leading to the Volga river, which entered the Caspian Sea, a route suitable for tanker travel during high water levels after the winter thaw. These tankers pioneered the method of carrying liquid cargo by ship.

In 1873, Robert Nobel, Alfred’s brother, traveled to Baku’s harbor with a mission to buy walnut wood for rifle butts, a task given to him by his brother Ludvig, who was in charge of the Nobel’s armament business in Russia. Robert, perceiving the potential of the new oil industry developing in Baku, used the money to buy a small oil field. Ultimately, Ludvig and Robert built an enormous oil company (Branobel) that produced and distributed over half the kerosene in the Russian Empire. Ludvig and Robert had the first oil tanker built, commissioned the Zoroaster, which plied the Caspian Sea (Figure 2).^14^,^15^ They also developed railroad oil tanker cars for transport over land and built one of the earliest oil pipelines that facilitated oil distribution. Ludvig Nobel was compared to Rockefeller, and by the beginning of the twentieth century Russian oil output exceeded that of the United States. A portion of Alfred Nobel’s wealth came from the family’s oil profits, principally the result of Ludvig’s and Robert’s efforts, espe- cially the technical and entrepreneurial skill of Ludvig.

In 1863, Alfred returned to Sweden to assist his father in the study of nitroglycerin, a volatile solution of nitric and sulfuric acid and gelatin, discovered in 1847 by Ascanio Sobrero, an Italian chemist, but not developed into an explosive. The material was exquisitely sensitive to jarring, and it could not be handled, manufactured, or transported safely. Indeed, Alfred’s younger brother, Emil Oskar Nobel, and a laboratory technician were killed in an accidental explosion in a Nobel laboratory. Within a year, Alfred produced a detonator for a large quantity of nitroglycerin or other explosive substance too dangerous for handlers to explode directly. Two years later, in 1865, he patented the mercury fulminate detonator. This percussion detonator was used to control the explosion of a large explosive mass, becoming the basis for most military and civilian blasting applications; it became known as the “Nobel lighter.”

Alfred found that kieselguhr (diatomaceous earth), a clay-like material composed principally of porous silica, absorbed nitroglycerin nearly to dryness (a thick paste) and made it insensitive to vibration, converting an unmanageable and dangerous material to one that could be handled with much less risk. The combination of nitroglycerin and diatomaceous earth, which Alfred named “Dynamite,” after the Greek word for power, “dunamis,” was one of his greatest technical achievements. Dynamite could also be shaped into cylinders that could be inserted in mining holes to expose deposits. Dynamite allowed construction projects to be carried out on a scale previously not possible. Blasting tunnels through mountains for a railway or a road, digging canals, and clearing navigation barriers from major rivers were some of the civilian applications of the new explosive. The relevant patents and licenses resulted in much of the large fortune that later formed the basis for the establishment of the Nobel Prizes.

Alfred held 355 patents for inventions in the field of industrial chemistry. He opened factories devoted to the manufacture of dynamite and, subsequently, its derivatives—blasting gelatin in 1875, more stable and powerful than dynamite, and, in 1887, ballistite composed of nitrocellulose and nitroglycerin, a substitute for black gun powder, the last-mentioned developed by the Chinese nearly a thousand years earlier. A variant of this explosive was used as a solid fuel propellant for rockets in the mid-twentieth century. He was able to secure a share of the profits from virtually every factory manufacturing explosives anywhere in the world.

In 1870, Nobel moved his headquarters and personal laboratory to Paris where he devoted his time to finding investment partners, plant locations, and managers for new factories, patent protection, and other business matters. In 1891 the business climate in France deteriorated, and Alfred moved his activities to San Remo, Italy where he worked until his death in 1896.

Nobel attached no consequence to personal honors. He trivialized his own receipt of the French Order, an irony in view of his proposal to use prizes to recognize and, presumably, to promote outstanding achievement. He was devoted to literature and considered leaving the family businesses for a career as a writer.^16^ By the time he left Paris for San Remo, his library contained over 1,500 volumes, many in their original languages. His collection was eclectic but was primarily fiction, including the works of nineteenth-century authors, and classical works, including the writings of Shakespeare and works on philosophy, religion, history, and science. He had a large collection of personal letters. He wrote numerous poems, the drafts of several novels, and the script of a play. In Paris he interacted with the literati and visited literary salons where he met contemporary writers and had a personal relationship with Victor Hugo, for whom he had particular admiration.

He never married; however, he did have a romance with Sofie Hess, an Austrian woman who was 23 years younger than he and whom he met in a shop in 1876 when he was 43. Another Austrian woman, Bertha Kinsky, became his confidant. She had responded to an advertisement in an Austrian newspaper placed by Nobel for a secretary and housekeeper. Bertha, then 33, traveled to Paris to be interviewed and was offered and accepted the position. Nobel became impressed with her intellect and interest in world affairs. Bertha soon left and married the son of a couple for whom she had worked previously and became the Baroness von Suttner. She and Nobel interacted again in 1887 when she became involved in efforts to bring peace to the countries of Europe**.** She published two important books. The first, *Die Waffen nieder*, later published in English under the title *Lay down your arms*, was an influential work*.* **S**he probably contributed to Nobel’s decision to establish a prize for the promotion of harmony among countries. She was awarded the Nobel Peace Prize in 1905 for her efforts to encourage peaceful relations among the nations of Europe. In an early version of his will, Nobel restricted the award of the Peace Prize to a period of 30 years. It was his opinion that if harmony among nations was not achieved in that time-frame, the world could not be saved from continued irrational, violent, and destructive relationships. His prediction was prescient.

## THE NOBEL FOUNDATION

In 1895, a year before his death, Nobel’s final will directed the establishment of the Nobel Foundation and a mechanism to select and fund the five Nobel Prizes. It took five years to make his ideas operational since he had no discussions about who would execute these plans and the specific mechanisms that would be used to select awardees. Nobel’s decision that Norway, not Sweden, award the Nobel Peace Prize was offensive to the Swedes, especially the royal family. Norway was seeking independence from Sweden, and their relationship was adversarial. The Swedish King interpreted Nobel’s will as an expression of support for Norway’s break from Sweden, and there was consideration of rejecting the will’s instructions to establish the Foundation.

The Nobel Foundation was established to manage the funds, supervise the selection of awardees, and organize the award of the prizes. Ragnar Sohlman, an engineer working in Nobel’s Karlskoga laboratory, and the Swedish industrialist Rudolph Lilljequist were the executors of his will. The potential Nobel heirs, notably two nephews, sons of his brothers, Robert and Ludvig, initially were distressed to find that only a very small fraction of the estate was to be divided between them and that they were not executors; they considered contesting the will. Another challenge came from Sofie Hess, who maintained that she was Alfred’s common-law wife and deserved inclusion in his bequest. The objections of the heirs were satisfied when one of the nephews reconsidered and facilitated discussions between the executors and the family. This nephew also helped resolve disputes having to do with the Russian oil field holdings, permitting an agreement that was acceptable to all segments of the Nobel family. Sofie Hess was given a lifetime allowance.

Problematic, initially, was the question of Alfred Nobel’s citizenship and the country to be the home of his fortune and Foundation. Nobel had left Sweden as a child and was not considered a citizen of that country nor, indeed, any other. Because of the international character of his businesses, it was not clear that Swedish courts had the jurisdiction to oversee liquidation of his properties and establish the Foundation. Disagreements arose with the French over which country should receive his estate. The determination of which country’s courts should adjudicate Nobel’s will and estate rested on an arcane French custom. According to French practice, a man’s residence was the place he kept his carriage horses. Nobel had moved his carriage horses to Sweden before his death. He was declared, ultimately, a legal resident of Sweden.

Two of the institutions entrusted with selecting recipients of the scientific prizes, the Royal Swedish Academy of Sciences and the Royal Caroline Institute (Karolinska Institutet), initially impeded the plan when they requested that a portion of the bequest be used to set up research institutes and to cover their expenses in selecting awardees.^17^ These requirements were met, allowing the implementation of the Prizes.

After all property was liquidated and debts paid, the amount available for establishment of the Nobel Prizes was more than 31 million kronar, equivalent to about $9 million United States dollars at the time or nearly $300 million in today’s dollars based on inflation, but would be considerably greater through investments. In accordance with the will, the funds were initially invested in “safe” securities, Swedish government bonds. This requirement retarded growth of the fund until its repeal in 1953.

On June 29, 1900, the Nobel Foundation was approved by King Oscar II and his cabinet. The Board of Directors of the Foundation consisted of five members charged with managing the investments and generating income for the prizes. The chairman of the Board was appointed by the Swedish King or Queen, and the remaining four members were designated by the institutions charged with selecting awardees.

Initially, the Nobel Foundation was required to pay taxes on its earnings. This requirement markedly reduced the net yearly income available for the prizes until 1946 when the Foundation was granted a permanent tax exemption. The Foundation published works about Alfred Nobel and the prizes, among them “Les Prix Nobel,” an annual series that includes biographies of the laureates and their Nobel lectures. These are now accessible on Nobel Foundation sites on the internet. It also publishes fact sheets giving the demographics of the prize winners by gender, age, country of origin, and other variables, also accessible on the internet.

The Foundation also provides funds for the expenses of the institutions and committees making selections for the awards, arranges travel for and organizes the yearly award ceremony, and hosts scientific symposia. In accordance with the operating statutes, separate committees were set up by the Swedish Academy of Science to select awardees for the prize in physics and in chemistry and by the Royal Caroline Institute to make the selection for the prize in physiology or medicine (Table 1). The Swedish Academy was selected to oversee the selection of the prize winner for literature “… of an idealistic tendency.” Apparently, translation from the Swedish leaves some ambiguity in the interpretation of “idealistic.” The literal interpretation of his instructions may have led to the omission of writers of singular note, such as Leo Tolstoy, and the selection of authors who have passed into obscurity. Two Nobel Laureates in Literature refused their award: Boris Pasternak, prevented by the Soviet Union from accepting it, and Jean-Paul Sartre, who did not accept awards as a matter of principle. The Nobel Foundation still recognizes awardees as Nobel Prize winners in their records whether they accept the prize or not.

**Table 1:** The Nobel Institutions Charged with Selecting the Laureates.

Prize Category	Year First Awarded	Responsible Institute
Chemistry	1901	Royal Swedish Academy of Science
Physics	1901	Royal Swedish Academy of Science
Physiology or Medicine	1901	Nobel Assembly at the Royal Caroline Institute (Karolinska Institutet)^*^
Peace	1901	Norwegian Nobel Committee
Literature	1901	The Swedish Academy
The Sveriges Riksbank Prize in Economic Sciences in memory of Alfred Nobel^**^	1969	Royal Swedish Academy of Science

* Composed of 50 professors at the Institute.

** Although not established as a Prize through Nobel’s will and the Nobel Foundation, it has been accepted functionally as “the Nobel Prize in Economics” and is often referred to in that way by the media.

The committees charged with selecting Nobel Laureates developed a set of procedures. In September of the year preceding the awards, requests for nominations are sent to members of the Institutions, professors at major Swedish and foreign universities and research organizations, and previous winners of the Nobel Prize. The nominations by those contacted must be made by the end of January in the year the award is to be made. Numerous requests for nominations are made, and these result in many candidates for each prize each year. Self-nominations are not accepted. The committees have the challenge of reducing a long list of qualified candidates to one, two, or, at most, three recipients for each prize. Evaluating the candidates and their accomplishments runs from February until the following September. Outside experts are consulted for advice on the importance of candidates’ achievements. In the committees’ deliberations, Nobel’s stipulation that the discovery for which the awards are given shall have conferred the greatest benefit on mankind is considered paramount. The recommendations of the committees are submitted to their institutions for approval. After approval by the institutions, the candidate for each prize is notified and announced to the public in October, the month of Nobel’s birth.

**Figure 3:**
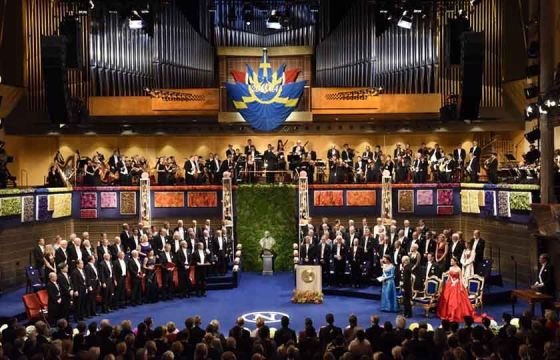
The Nobel Prize Ceremony in 2014 with the Laureates in the Front Row on the Left and the Royal Family on the Right. In this year, the Prize in Physiology or Medicine was shared by John O’Keefe, May-Britt Moser, and Edvard I. Moser “for their discoveries of cells that constitute a positioning system in the brain.” Photograph taken by Niklas Elmehed. ©Nobel Media AB. Permission obtained from the Nobel Foundation.

**Figure 4:**
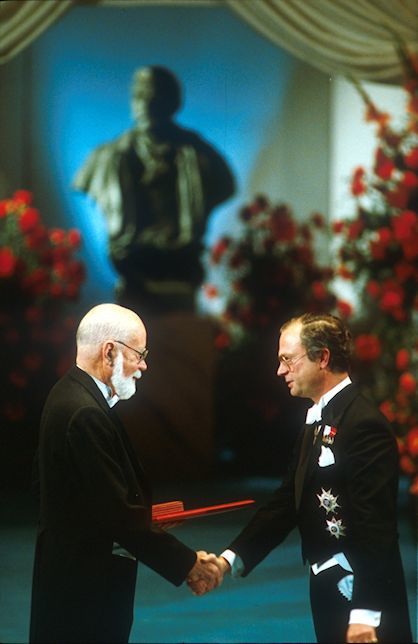
Edward Donnall Thomas Receives his Medal and Certificate for the 1990 Nobel Prize for Physiology and Medicine from His Majesty Carl XVI Gustaf, King of Sweden. Thomas received the prize for the development and clinical application of hematopoietic stem cell transplantation. The prize was shared with Joseph Murray for his development of renal transplantation. Image provided by the Fred Hutchinson Cancer Center, Seattle, WA.

The announcement of the recipients of the Nobel prizes in October receives more attention from the worldwide media than the formal award ceremony in the Stockholm Concert Hall on December 10, the anniversary of Nobel’s death. The ceremony is presided over by the King and Queen and is attended by an audience of as many as 1,000 persons. Figure 3 depicts a Nobel ceremony with laureates and royal family in apposition. Figure 4 shows E. Donnall Thomas receiving the Nobel Prize in Physiology or Medicine in 1990 from His Majesty Carl XVI Gustaf for his singular contribution to the development of syngeneic and allogeneic hematopoietic stem cell transplantation, commonly referred to as bone marrow transplantation.

**Figure 5:**
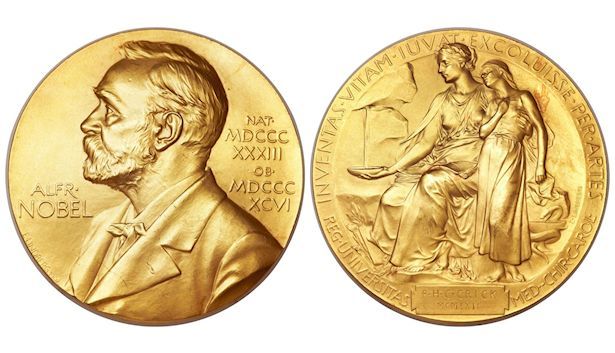
The Medal for the Nobel Prize in Physiology or Medicine. A portrait of Alfred Nobel is on one side of the medal for each prize. The opposite face is uniquely relevant to the discipline for which each prize is awarded. This medal depicts the “genius of medicine” represented as a woman seated with an open book on her lap. The woman is filling a bowl of water from a spring to relieve a suffering girl’s thirst. There is a Latin inscription above: “Inventas vitam juvat excoluisse per artes” (“Invention enhances life, which is beautified through art”) cited from Virgil’s Aeneid. The name of the laureate is engraved on the plate below the figure, in this case Francis Crick, the co-discoverer of the structure of DNA. The lower text “REG. UNIVERSITAS MED. CHIR. CARO.” designates the Royal Caroline Institute (Karolinska Institutet). Reproduced with permission of the Nobel Foundation. © ® The Nobel Foundation.

The Nobel Prize consists of a gold medal, a unique diploma fashioned by an artist citing the reasons for the prize, and the monetary award. The medal was struck originally from 23-carat gold. Since 1980, the medal is struck in 18-carat green gold with 24-carat gold plating. The medal given for the Nobel Prize in Physiology or Medicine is shown in Figure 5. The medal for each prize has a different image on the back relevant to the field of achievement. The medal for economics has a different image of Nobel on its face and different text including the designation of the Bank of Sweden. The monetary amount distributed with each prize varies and depends upon the income available from the Nobel Foundation’s investments. In its first years, the monetary award was equivalent to approximately 20 years’ salary for the average university professor. As such, it was a lifetime stipend to permit the awardee to continue his or her work without concern about funding. By the 1990s each prize was valued at over one million dollars.

**Figure 6:**
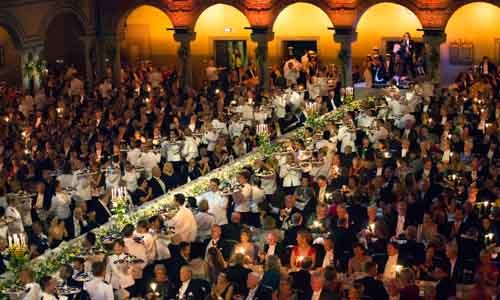
The Nobel Prize Banquet in 2013. In this year, the Nobel Prize in Physiology or Medicine was shared by James E. Rothman, Randy W. Schekman, and Thomas C. Südhof “for their discoveries of machinery regulating vesicle traffic, a major transport system in our cells.” Over 1,000 attendees are accommodated, requiring military precision in delivering the multicourse dinner in a tasteful and efficient manner. Photograph taken by Alex Ljungdahl. ©Nobel Media AB. Permission obtained from the Nobel Foundation.

Following the awards, a lavish banquet is held in the Blue Hall of the Stockholm City Hall. The size of the banquet has grown ten-fold since 1901 to accommodate over 1,000 persons who attend (Figure 6). The menus and dinner courses, the place settings of flatware and china, and the logistics of serving the large assembly of laureates, their family and friends, the royal family, distinguished guests including members of the selection committees and the Nobel Foundation, diplomats, government officials, and others have been the subject of various writings, including a monograph.^18^ Table 2 provides an example of the elegant menu, in this case for the dinner in December 2016.

**Table 2:** The Nobel Banquet Menu, 2016.^19^

Charcoal baked langoustine and scallop, served with nettles, ramson and pickled winter apples
Quail from Södermanland in black garlic and leek ash with Jerusalem artichoke, preserved wild mushrooms and jus of roasted chicken skin and mustard seed
Cloud of sudachi fruit, cloudberry sorbet, miso crumbs and deep-fried rice paper
**Wine**
Taittinger Comtes de Champagne Brut Blanc de Blancs 2006
Piccini Poggio Teo Chianti Classico 2010
Moncaro Tordiruta Passito 2007
**Coffee & Nobel Museum Tea Blend**
Grönstedts Extra Cognac
Facile Punsch
Stenkulla Brunn Mineral Water
Stadshusrestauranger in collaboration with Chef Sayan Isaksson as well as Pastry Chef Daniel Roos
The menu is in French with an English and Swedish translation.
© ® The Nobel Foundation.

The Nobel Foundation has allowed selection committees to deviate from Nobel’s stipulation that the achievement for which the prize is awarded be in the preceding year. The committees have given prizes for discoveries made decades before the year of the award. Penicillin, for example, was discovered by Alexander Fleming in 1928 and developed into a drug by Howard Florey and Ernst Chain in the early 1940s, and the award was given to the three in 1945. Peyton Rous, the oldest awardee, received the prize in 1966 at age 87 for work reported in 1910, 56 years earlier, on a transmissible (viral-induced) sarcoma in fowl. The Nobel Prize, especially in Physiology or Medicine, has in recent decades more often been given as an award shared by two or three scientists. The Nobel Foundation has agreed that no more than three awardees can share a single prize. Each awardee receives his or her medal and diploma; however, the monetary award is divided equally among them.

## THE NOBEL PRIZE IN PHYSIOLOGY OR MEDICINE

The Nobel Prize in Physiology or Medicine, because of its relationship to human health and well-being, is the one that can be identified most readily by the public as meeting Nobel’s intent to recognize the accomplishment that is a benefit to mankind. The prize in physiology or medicine has not been awarded every year. For example, no prizes were offered for several years during the First and Second World War and in several years in which an appropriate candidate was not identified. From 1901 to 2016, 107 awards have been made to 210 laureates, of whom 12 were women.^20^,^21^ Approximately one-third of the prizes were given to one laureate, one-third was shared by two awardees, and one-third was shared by three awardees. The Nobel Prize in Physiology or Medicine has been awarded without interruption from 1943 to 2016.

Prior to World War II, Europe was the center of scientific research, and European scientists received most of the prizes for physiology or medicine. From 1901 to 1939, only three prizes for physiology or medicine were won or shared by North Americans: the first to Canadians, Frederick Banting and John James Rickard Macleod, for the isolation and clinical use of insulin in 1923; the second to Thomas Hunt Morgan for his early studies of genetics and inheritance, pioneering the use of *Drosophila melanogaster* (the fruit fly) for genetic research; and the third in 1934, for the cure of pernicious anemia, was shared by George Richards Minot, William Parry Murphy, and George Hoyt Whipple, to be discussed subsequently in this paper.

Following the Second World War, with the massive disruption and destruction in Europe and the establishment of the National Science Foundation and the expansion of the National Institutes of Health, the United States became the home of the most generously supported and largest biomedical research establishment in the world. From 1943 until the current time, over 70% of the laureates in physiology or medicine have been either native or naturalized US citizens.

In the pre-World War II period, 42 of the 45 laureates in physiology or medicine held a medical degree, and many practiced medicine. The Nobel Prize in Physiology or Medicine reflected successes in the late nineteenth and early twentieth century in conquering diseases caused by microbes and elucidating major physiologic functions of the body. Robert Koch, Paul Ehrlich, Élie Metchnikoff (one of only two Russian, more specifically Ukrainian, laureates in physiology or medicine), Jules Bordet, Ivan Pavlov (the other Russian), and Karl Landsteiner are only a few of the laureates on the list, which is a treasure trove of great European physicians of the late nineteenth and early twentieth century.

Following the Second World War, the awards began to focus on fundamental biochemical or molecular discoveries—including such areas as the physical and chemical basis of nerve conduction, the chemical basis of vision, investigations of tumor viruses, the genetic basis of atherosclerosis, the action of cell growth factors, the cellular origin of cancer genes, the fundamentals of bacterial or viral genetics, DNA replication in bacteria, the structure of DNA, the biosynthesis of DNA and RNA, pluripotential stem cell biology, basic elements of the immune system, and so on—making the direct benefit to mankind demanded by Nobel’s will less apparent to the lay observer and certainly less immediate.

Despite the focus on basic discoveries, at least two awards in the modern era were made to practicing physicians: the 1990 prize shared by Edward Donnall Thomas, an oncologist, and Joseph Edward Murray, a transplant surgeon, for the development of hematopoietic stem cell transplantation and renal transplantation, paving the way for liver, heart, lung, and other solid organ transplantation. The 2005 award was shared by a clinical pathologist, John Robin Warren, and an internist-gastroenterologist, Barry James Marshall, for the discovery of *Helicobacter pylori* and its role in gastric inflammation and peptic ulcer development. Later the organism was shown to cause gastric carcinoma and gastric mucosa-associated lymphoid tissue lymphoma.

One Nobel Prize in Physiology or Medicine was awarded for what proved to be erroneous research, the award in 1926 to Johannes Andreas Grib Fibiger, a Danish professor of pathological anatomy, who reported the discovery of a worm that caused cancer of the rat stomach, which he designated *Spiroptera carcinoma*. The lesions were later shown to be hyperplastic, not neoplastic.^18^ Remarkably, three reports that were contemporaneous with Fibiger’s showing that coal tar produced cancer of the skin of animals; that a putative cancer virus could transmit fowl tumors; and that *Schistosoma haematobium* infection can result in bladder cancer were overlooked.^22^ The discovery of a transmissible agent that caused fowl sarcoma by Peyton Rous was recognized by the Nobel Foundation approximately a half century later. Each of these three mechanisms of cancer initiation has stood the test of time.

A second prize may have been premature, and certainly controversial, namely the 1949 Nobel Prize in Physiology or Medicine to António Egas Moniz, a Portuguese neurologist, for the treatment of severe psychiatric disorders, especially schizophrenia, by prefrontal lobotomy.^23^ The prize was shared with Walter Rudolf Hess for his neurophysiological studies of the diencephalon. A notable victim of the lobotomy procedure was Rosemary Kennedy, sister of US President John Fitzgerald Kennedy, an attractive, interactive woman, whose functional state was converted to a vegetative state by the procedure inflicted on her at the insistence of her father in 1941.

Today, with a research establishment that spans the world and with several hundred accomplished nominees each year, it is very difficult to award the Nobel Prize in Physiology or Medicine to the most deserving scientist or physician. Indeed, laureates recognize that their work would not have been possible without the discoveries of other scientists whose work had not been recognized by the Foundation.^24^ A former chairman of the Nobel Foundation, Arne Tiselius, himself a laureate, in response to a query about how laureates are selected, indicated that one cannot in practice apply the principle that the Nobel Prize should be given to the person who is best; it is impossible to define who is best. Hence, there is only one alternative: to try to find a particularly worthy candidate.

The Nobel Prize in Physiology or Medicine awarded in 1934 for the treatment of pernicious anemia holds special significance for the University of Rochester School of Medicine and Dentistry, which was nine years old in 1934, the first post-Flexnerian medical school established in the United States, when its founding Dean, George Whipple, shared the prize for his work on the repair of anemia in chronically bled dogs and the importance of liver in the diet to repair the anemia most efficiently.^25^

In the late nineteenth and early twentieth century, patients in North America and Europe with an eventually fatal type of severe anemia were being described. The affected patients also had severe neurologic damage, and the outcome of the disease, although more protracted, was similar to adults with acute leukemia, invariably resulting in death. The patients often had pancytopenia, profoundly proliferative and dysplastic marrow cells, and neurological impairment. In 1908, Richard Cabot of Boston provided a comprehensive clinical description of the disease and an analysis of 1,200 patients. He found that survival after onset was 1 to 3 years.^26^ The anemia was referred to as “pernicious.”

In 1918, Whipple began his experiments on dogs bled to half normal hemoglobin levels. A basal diet of canned salmon and bread allowed periodic withdrawal of blood to maintain the low blood hemoglobin. If a diet was introduced that contained beef liver or muscle, hemoglobin production increased. Whipple and his co-worker, Frieda Robscheit-Robbins, published a paper in the *American Journal of Physiology* on blood regeneration and severe anemia in the 1920s that highlighted the favorable influence of liver in the diet on the regeneration of red blood cells in their dog model of anemia. They stated: “Liver feeding in these severe anemias remains the most potent factor for the sustained production of hemoglobin and red cells.”^25^ In publications in 1922 and 1925, Whipple encouraged physicians to consider dietary factors in the management of anemic patients based on his studies in dogs.^25^

In 1925, William Murphy had one year earlier gone into medical practice and was on the staff of the Peter Bent Brigham Hospital in Boston. He agreed to work with George Minot, who was a physician-investigator on the faculty of the Harvard Medical School and the Huntington Memorial Hospital, on a project to determine if a form of diet therapy could help patients with pernicious anemia, a disease in which Minot had a special interest. Minot’s view that a dietary factor may play a role in the development of pernicious anemia grew out of the notion that “good food makes good blood,” which was a general theme during the late nineteenth and early twentieth centuries. In 1926, Minot and Murphy astounded the world of medicine with the announcement at the meeting of the Association of American Physicians at its annual gathering in Atlantic City that they had cured the anemia in a series of 45 patients. These patients had been fed a special diet that contained up to one-half pound of lightly cooked beef liver, daily, for several months. This was an unappetizing diet, especially in patients seriously ill with loss of appetite and other gastrointestinal disturbances. Nevertheless, the effect of this discovery was not only to reverse the death sentence for these patients but to encourage and stimulate research concerning diseases of the blood.

Minot credited Whipple for highlighting the importance of nutrition as a potential factor in anemic patients and for focusing on liver. In their paper in the *Journal of the American Medical Association* in 1926, Minot and Murphy reported the salutary effects of liver feeding in pernicious anemia patients. Minot’s and Murphy’s paper had several references to Whipple’s and Robscheit-Robbin’s work and one reference to their own prior work, although none of these prior reports had anything to do with dietary treatment of pernicious anemia.^27^

Some observers felt that Whipple’s role was not consequential enough to merit his sharing the prize. In a monograph entitled *Anemia in practice: Pernicious anemia*, written by Murphy and published in 1939, he wrote in his chapter entitled “The Introduction of Liver Therapy” that,

It became our task then to prove the practicability of an idea which had up to this time received no intensive study or definite confirmation. Some years previously Whipple and his co-workers had demonstrated that liver had a rather unusual value for the production of hemoglobin in dogs made anemic by bleeding. Because this worked entirely with the production of hemoglobin as opposed to the maturation of erythrocytes, the latter being the problem chiefly concerned in recovery in pernicious anemia, because it was carried out entirely on animals, rather than human beings, and because the results of the study had no bearing upon or reference to pernicious anemia as observed in mankind, the study which we were to undertake was of a pioneering nature. No background evidence proved that it would be beneficial, except that a few patients who had been advised by Dr. Minot to ingest some liver together with red muscle meat, as part of their diets, had apparently remained in better health during short periods of time, than those who had not used liver.^28^

Whipple was studying the response of iron deficiency anemia produced by chronic bleeding of his dogs, whereas Minot and Murphy were later shown to be studying vitamin B12 deficiency. Liver was a rich source of iron and vitamin B12 and, thus, reversed the iron deficiency anemia in Whipple’s dogs and the anemia in humans with pernicious anemia by happenstance and for quite different reasons. Minot and Murphy did not know the pathogenesis of pernicious anemia, only that something in liver could reverse its expression. In the context of the late 1920s and early 1930s, this finding justified their selection. They cured a fatal disease. The reason liver feedings worked was that the daily requirement for vitamin B12 is minuscule, approximately one millionth of a gram per day. The vitamin B12 contained in liver is very large in comparison, and sufficient liver was eaten by patients to provide this minuscule amount of vitamin B12 across the intestinal wall by mass action in the absence of intrinsic factor in gastric juice, normally required for vitamin B12’s absorption.

In 1927 and 1928, a Harvard physician-scientist, William Castle, published a series of ingenious experiments in humans showing that a missing factor, secreted by the stomach, which he referred to as an intrinsic factor, was necessary for the absorption of something from food sources to maintain blood production and nervous tissue integrity, including the brain and spinal cord. The factor in food was referred to as the extrinsic factor, later found to be vitamin B12. It was much later determined that pernicious anemia was an autoimmune disease in which an autoimmunological attack was directed at the stomach lining cells leading to gastric atrophy and the inability to secrete hydrochloric acid and intrinsic factor, normal constituents of the gastric juice. Intrinsic factor is required to complex with vitamin B12 from food sources to permit that vitamin’s absorption in a specific area of the terminal small intestines. Some thought Castle should have shared the Nobel Prize with Minot and Murphy, not Whipple. Vitamin B12 was characterized in 1948 and intrinsic factor in 1961.

Minot had become a severe diabetic as a young adult. He was cared for in Boston by Elliot Joslin with a severely restricted sugar diet. Joslin later founded the Joslin Clinic, a pioneering institution for diabetic care. Minot was alive to make this contribution because of the discovery of insulin in 1922, for which the Nobel Prize in Physiology or Medicine was awarded in 1923 to Frederick Banting and John Macleod. The omission of Charles Best, Banting’s colleague in the laboratory, who was integral to the research as one of the awardees in 1923, angered Banting. This dispute was yet another controversy over a Nobel selection decision. Banting shared his monetary award with Best. Because of the long and arduous sea voyage to Stockholm, and an unfamiliar medical environment, a physician accompanied Minot to administer insulin, assist in his rigid low-sugar diet, and to attend to his diabetes, should it go out of control. This medical support allowed him to accept the prize in person, generally a requirement of the Nobel Foundation for receipt of the prize.

Many deserving scientists have been overlooked, too numerous to cite. The so-called forty-first chair is so designated because of the 40 places available in the French Academy; it represents the deserving scientists who just missed selection. For a short period after the establishment of the prizes, the Nobel Foundation published the names of runners-up or honorable mentions, but they stopped doing so. These holders of the forty-first chair include among them people who should have won the prize, but did not, in some cases because they died before their work was recognized as ground-breaking. Since 1974, a Nobel Prize may not be awarded posthumously. Only two prizes were awarded posthumously prior to 1974: one for peace and one for literature. One prize in physiology or medicine was made posthumously because the recipient, Ralph Steinman, working at the Rockefeller University, who discovered the dendritic cell and its function in the immune system, died a few days before the announcement of his selection in October 2011, unbeknownst to the Nobel Foundation. Having made the announcement not knowing he had died, the Foundation proceeded to make the award, posthumously, in that December.

One of the most notable omissions in the Nobel Prize for Physiology or Medicine was Jonas Salk.^29^ In 1955 the announcement was made that polio was conquered as a result of the singular efforts of Salk to develop, manufacture, and field test the first vaccine. Polio was a scourge in most industrialized countries. In the United States, summers were periods of terror for parents who often kept children from contact with playmates in camps, swimming pools, or sharing a water fountain, and applied other restrictions in the hopes of minimizing viral transmission. The vaccine made unnecessary the need to warehouse the numerous “iron lungs,” respirators used to ventilate children and young adults with “bulbar” polio, previously stored at the ready in most major hospitals for the summer polio season. In July 1960 one iron lung remained at the Strong Memorial Hospital, the University of Rochester Medical Center; it was used it to manage a young man with Guillain–Barré syndrome with respiratory muscle paralysis.

Salk’s work was viewed as applied and only a technical achievement (low-brow) by some of his influential peers. Perhaps, it was not “high science,” but who better would have fit Nobel’s desire to give the prize to someone who benefited mankind in the previous year? This achievement was among the most impactful on human health in the last 70 years. Salk was neither elected to the National Academy of Sciences nor to the American Philosophical Society, although the membership of both organizations included the country’s leading medical investigators.

Some Nobel Prizes in Physics or in Chemistry honored discoveries that later were seen to be important basic contributions to medicine. One of the most important was the initial Nobel Prize in Physics in 1901 given to Wilhelm Roentgen for his discovery of a new form of ray, which he called X-rays, using the mathematician’s symbol “X” for its then unknown properties. Few discoveries have had such a profound and lasting impact on medical diagnosis and therapy.

In 1962, Francis Harry Compton Crick, James Dewey Watson, and Maurice Hugh Frederick Wilkins shared the Nobel Prize in Physiology or Medicine for the elucidation of the structure of DNA. Another controversy in the selection of awardees surrounded the omission of Rosalind Franklin whose crystallographic images of DNA were instrumental in deducing its structure. Her X-ray diffraction pictures of DNA taken in a different physical state, not strictly crystalline, established the helical structure of the molecule and that its diameter indicated that it must be made of two intertwined chains. No scientist had imagined DNA had a multi-chained structure. She also corrected the erroneous belief that the phosphate-sugar backbone was in the interior of the molecule with the bases pointing outward. This information was essential for Watson and Crick to build their 3-dimensional model that met the most satisfactory configuration. Their landmark paper was published in the journal *Nature* in April, 1953, immediately followed in that issue by a paper by Wilkins and another by Franklin. They summed to confirm the double helical structure of DNA, each strand bound together by specific base pairing of nucleotides: adenine and thymine or cytosine and guanine. The paper by Watson and Crick was one page long, a model of brevity in scientific exposition. In retrospect, their paper may have been the most important contribution to the life sciences since Darwin’s book *On the origin of species* in 1859, 94 years earlier. Yet, it took the Nobel Foundation nearly a decade to honor its significance, by which time Franklin had died of ovarian cancer. She had not been nominated during her lifetime.

In 2013, 51 years after the award, the family of Crick, by then deceased, sold his Nobel Prize medal at auction to the CEO of a Chinese biomedical firm for over two million dollars.^30^ A seven-page letter by Crick to his 12-year-old son, explaining his discovery, which pre-dated the publication of the seminal paper in *Nature*, was sold to an anonymous buyer for over six million dollars, exceeding by two-fold the previous highest price paid for a letter of historical importance, one written by Abraham Lincoln opposing slavery.^31^ A year and a half later, James Watson sold his Nobel Prize medal for over four million dollars.^32^ The proceeds of both sales were intended to be shared with academic institutions important to the careers of both scientists.^30^,^32^

## END NOTE

The remarkable persistence of the Nobel Prize as a revered indicator of achievement is a testament to Alfred Nobel’s stipulations and fortune and the Nobel Foundation’s adherence to the highest standards of selection. The prizes remain a measure not only of outstanding individual achievement, but, secondarily, of the ability of a country to provide the environment that permits the free and unencumbered pursuit of truth. The United States has benefited by (1) immigration to this country of outstanding scientists seeking such an environment in which there is scant religious or political pressure on research directions and outcome, (2) the wholesome and fulsome support of research with federal dollars through the National Science Foundation and the National Institutes of Health, and (3) the competitive national process that objectively determines which proposed research has the most sophisticated, insightful, and farsighted scientific ideas for grant awards. Large countries like the former Soviet Union, the current Russian Federation, the People’s Republic of China, other Asian and African countries (e.g. Iran, Indonesia, Pakistan, and Egypt) with people of genius have been underrepresented in awards. It may be that societal and cultural circumstances unfavorable to the support of strong scientific centers, unfettered scientific discovery, and space for unpopular or paradigm-breaking ideas are at play. An analysis has indicated that cultural and familial factors, such as the Judaic tradition of scholarship, may play a role in the development of scientists and writers capable of achievements that benefit mankind, Nobel’s quest.^33^ Over one-fifth of all Nobel laureates and one-third of all US laureates have been Jewish. Jews represent 0.2% of the world’s population.^33^ One can ponder and lament how many laureates were lost in Nazi concentration (death) camps among the six million murdered Jews and their descendants.

## Abbreviations

DNA: deoxyribonucleic acid
RNA: ribonucleic acid.
